# MIML: Multiplex Image Machine Learning for High Precision Cell Classification via Mechanical Traits within Microfluidic Systems

**Published:** 2023-09-15

**Authors:** Khayrul Islam, Ratul Paul, Shen Wang, Yaling Liu

**Affiliations:** a Lehigh University, Mechanical Engineering, Bethlehem, 18015, USA; b Lehigh University, Bio Engineering, Bethlehem, 18015, USA

## Abstract

Label-free cell classification is advantageous for supplying pristine cells for further use or examination, yet existing techniques frequently fall short in terms of specificity and speed. In this study, we address these limitations through the development of a novel machine learning framework, Multiplex Image Machine Learning (MIMLThis architecture uniquely combines label-free cell images with biomechanical property data, harnessing the vast, often underutilized morphological information intrinsic to each cell. By integrating both types of data, our model offers a more holistic understanding of the cellular properties, utilizing morphological information typically discarded in traditional machine learning models. This approach has led to a remarkable 98.3% accuracy in cell classification, a substantial improvement over models that only consider a single data type. MIML has been proven effective in classifying white blood cells and tumor cells, with potential for broader application due to its inherent flexibility and transfer learning capability. It’s particularly effective for cells with similar morphology but distinct biomechanical properties. This innovative approach has significant implications across various fields, from advancing disease diagnostics to understanding cellular behavior.

## 1 Introduction

Identifying and sorting target cells from heterogeneous populations constitutes a crucial initial step in numerous biological, biotechnological, and medical applications^1,2^. Following sorting, these cells may undergo detailed analysis, probing their proteomic, transcriptomic, or genetic identities and functions^3–5^. Alternatively, they can be utilized for regenerative medicine applications, such as transplantation into patients^6,7^. Cell sorting is traditionally performed based on molecular labels^8–10^. However, sorting methods leveraging intrinsic properties, such as cell size or deformability, have also been demonstrated^11–13^.

Nonetheless, it is imperative to acknowledge the limitations that emerge from the current approaches. While the high accuracy resulting from fluorescent methodologies is obtainable, it is not without its drawbacks^14–18^. The process of fluorescence labeling is both time-consuming and costly. Furthermore, fluorescent markers can interfere with cellular function and physiology, potentially altering their natural states and behaviors, thereby compromising the integrity of the research results[see supplementary note 2 for drawbacks of fluorescent-based sorting]. Particularly when applying label-free bright field imaging techniques for cell detection, a decrease in classification accuracy is observed, especially concerning visually indistinguishable cells. Recently, machine learning has begun to significantly reshape the contours of biomedical imaging, introducing a revolutionary level of precision and analytical depth^19–25^. The majority of machine learning-based on cell classifications have traditionally hinged on either image-based methods or the extraction of specific cell features^26–32^. Only a handful of studies have explored the intersection of label-free cell classification and machine learning^19,33,34^. This emerging field is crucial for developing refined cell classification methods for visually indistinguishable cells. Addressing this need has the potential to transform our understanding of cellular dynamics. An advanced, label-free classification could significantly advance cell biology and enhance biomedical imaging, paving the way for less invasive studies and new breakthroughs in cell classification.

In our endeavor to refine cell classification methodologies, we developed a novel machine learning architecture called Multiplex Image Machine Learning (MIML). This architecture is specially crafted to categorize visually similar cells, leveraging both label-free bright field images and intrinsic cellular mechanical properties as primary input features. The significance of including mechanical properties lies in their inherent ability to reflect cellular behaviors and states. MIML is implemented in a use case involving the classification of Tumor cells (HCT116s as an example) and White Blood Cells (WBCs) achieving a significant accuracy of 98.3% with a substantial improvement of *~* 8% over pure image-based classification. With these insights and results in place, the core achievements of our study are summarized in the following research highlights:

1. Architecture: An entirely new machine learning architecture is designed and introduced. This framework uniquely combines both image and cell mechanical properties to predict tumor cell types with high accuracy. Such an approach circumvents the necessity for fluorescent labeling, thus eliminating the associated drawbacks.
2. Interpretability: Through comprehensive feature analysis and activation layer visualization, we have achieved an interpretable AI model for tumor cell analysis. This clarity facilitates a deeper understanding and yields higher accuracy in cell classification.
3. Transfer Learning: Our research successfully demonstrated a novel approach that utilizes a combination of cell images and distinct mechanical properties for the classification of cells that are visually indistinguishable. The potential applicability of this methodology is extensive, and it could be generalized to classify an array of cell types that, despite exhibiting visual similarities, bear distinguishable cellular properties.

## 2 Results and Discussion

**Fig. 1:**
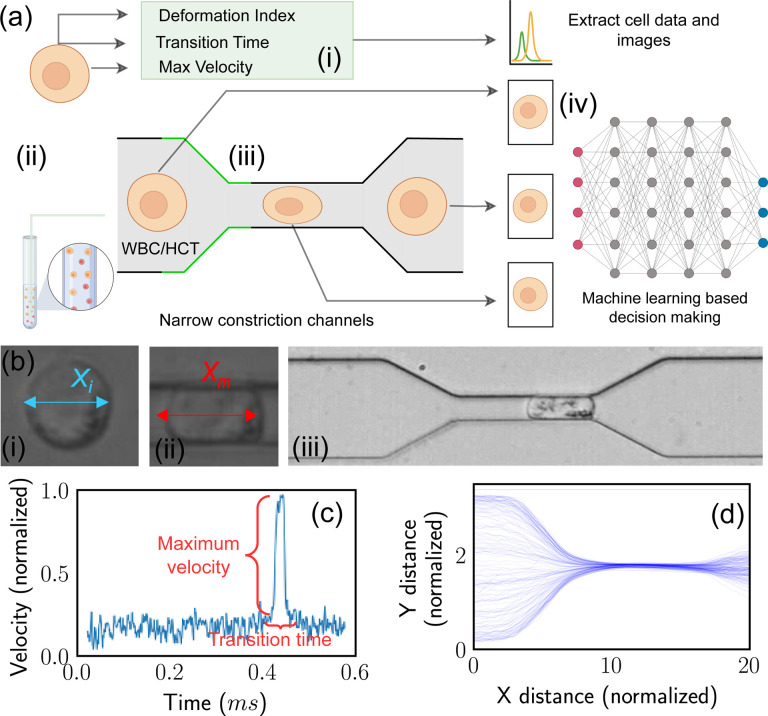
MIML Inferencing Process and Cell Analysis, (a) Schematic of the cell data collection and subsequent classification, (i) Biomechanical data collection, (ii) Preparation of cell samples, (iii) Cell transition through a narrow channel, (iv) MIML inferencing using cell imagery and textual data, (b) Experimental cell imagery, (i) Cell length prior to compression, (ii) Cell length while being compressed, (iii) Snapshot of a cell positioned centrally within the narrow channel, (c) Temporal velocity profile of cells, (d) Normalized cell progression through the squeezing channel.

The schematic representation of the standard procedure for cell detection via MIML is embodied in Figure 1(a). The subsequent sections are dedicated to a comprehensive examination of various classification models apt for numerical data. We also delve into traditional Convolutional Neural Network (CNN) models designed for image classification. Finally, we introduce our custom MIML model. The MIML model implements an innovative approach to predict cell type. It efficiently unifies images captured in real time as cells traverse through a narrow channel. The mechanical properties of these cells are evaluated through image processing techniques. This combination of visual and mechanical data allows for a more accurate and nuanced understanding of the cell type, thereby elevating the performance of cell detection and classification to unprecedented levels.

The effectiveness of the MIML model is demonstrated through an example application of differentiating tumor cells from white blood cells. Detection of circulating tumor cells in blood samples is important for early cancer diagnosis and monitoring of tumor progression^35–37^ [cite a few papers, including ours]. Label-free detection of circulating tumor cells without fluorescence labeling and antibodies is gaining popularity because they can be applied to a wide range of cancer types without a tedious labeling process^31,38,39^ [cite a few papers, including ours]. In this paper, we used a model circulating tumor cell sample by mixing HCT116 cells with white blood cells. HCT116 cells are a commonly used human colon cancer cell line, derived from a patient with colorectal carcinoma. Their genomic stability and well-characterized nature make them pivotal in oncological research. The goal is to demonstrate that a combination of image features and mechanical features in an integrated machine learning framework leads to higher classification accuracy than any of these features alone.

### 2.1 Composition of training and validation sets

In our study, we utilize two distinct forms of data for the purpose of training our models. The first, textual data, is provided in a CSV format and serves as the training dataset for our classification models. The second, image data, is deployed in the training of our CNN model. In the interest of robust evaluation, the data is partitioned into three distinct sets, namely, training, testing, and validation. The role of the testing data is to continuously assess the model performance throughout the course of training, whereas the validation data is reserved for an evaluation subsequent to the completion of the training process. This approach is pivotal in directing our model to recognize critical parameters, thereby aiding in the attainment of enhanced validation performance. Additionally, this method serves as a preventive measure against potential overfitting that comes from model learning parameters that result in high performance on the training and testing data but fail to generalize in overall application^40,41^

**Fig. 2:**
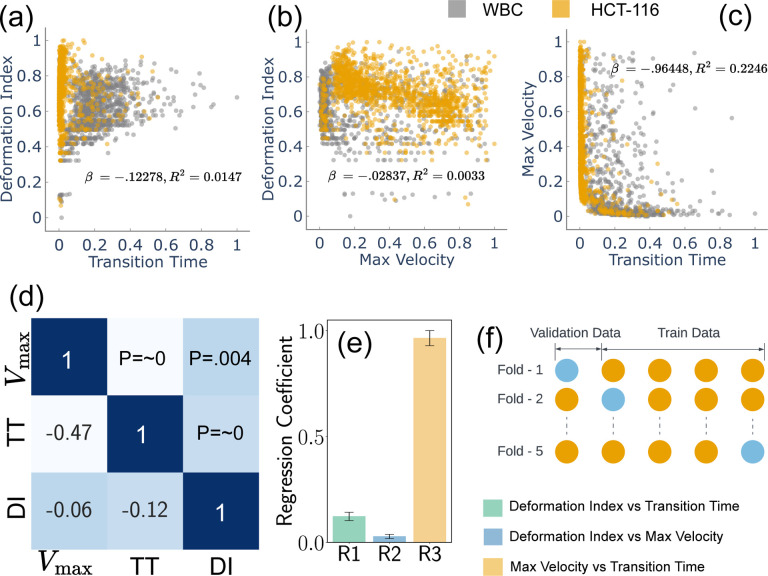
Examination of cellular biomechanical properties, (a-c) feature scatter plots that demonstrate the variability of inherent cellular attributes, (d) illustration of correlations between various features, as portrayed in a heatmap, accompanied by associated p-values (f) cross-validation utilized in this study, illustrating a scenario with four stacks used for training and one stack reserved for testing.

To get the mechanical properties of the cells, we have designed and fabricated a microfluidic device with a narrow channel smaller than the cell size. While the cells pass through such narrow channels, they will experience large deformation, leading to different translocation speeds and times. For each individual cell navigating through the narrow channel, we have captured two images - one at the beginning and another at the termination of the squeezing process 1(b)(iii). These images serve to train our CNN model, while an additional training data point per cell is employed for training our classification models. The total training data comprises 2521 entries, of which 1156 correspond to White Blood Cells (WBC) and 1365 to HCT-116 cells. For the purpose of training the CNN model, we possess a total of 5042 images, segregated into 2312 images of WBC and 2730 of HCT-116. The data corpus has been partitioned into training and testing sets with a 4:1 ratio. The larger portion (80%) is allocated for training and validating purposes, while the remainder (20%) is set aside for testing. This training set is further subdivided into five subsets for the implementation of cross-validation. During each iteration of the cross-validation, one subset is reserved for validating while the remaining four subsets are employed for training Figure 2(f). This meticulous data partitioning and utilization strategy underpins our methodical approach toward robust model training and performance validation.

### 2.2 Detection of cell classes by classification model

Image-derived metrics such as deformation, defined as the divergence from a perfectly circular shape, offer insight into the mechanical properties of the objects being measured^42,43^. This deformation, coupled with maximum velocity and transition time, emerges as a crucial parameter set for effective cell classification. We analyzed three distinct features of a cell navigating a narrow channel, namely, the cell’s deformation Index (*DI*), the Transition Time (*TT*) taken by the cell to traverse the narrow channel, and the maximum velocity *v*_max_ achieved by the cell within the channel’s narrow region1(b)(iii). The DI of the cell is quantified using the following formula:

(1) $$DI=\frac{\left(a-b\right)}{\left(a+b\right)}$$

In this equation, *a* and *b* represent the major and minor axes of the considered cell, respectively. The deformation index adopts a scale from 0 to 1, with 0 representing a flawless circle, thus indicating no deformation, and 1 corresponding to the utmost conceivable deformation, given an assumed minor axis of zero^44^. As the deformation index values come pre-normalized, it is crucial to extend this normalization to the remaining pair of features - transition time and maximum velocity. By doing so, we amplify our model’s generalization potential and encourage its adaptability across different contexts through transfer learning. Therefore, we ensure these features also conform to a 0–1 scale and proceed to scrutinize their correlation.

Figure 1 provides a detailed representation of the data acquisition methodology, complemented by the microfluidic channel employed for cellular deformation. The velocity, as delineated in 1(c), is normalized in accordance with the flow velocity. Meanwhile, Figure1(d) depicts the position of the cell’s centroid, which is presented in values normalized relative to the cell’s dimensions. This figure effectively captures the trajectory of the cell within the microfluidic channel. Figure 2(a–c) illustrated scatter plots capturing the relationship between each pair of feature sets under study. A notable observation from Figure 2(a) is that HCT-116 cells consistently exhibit shorter transition times and greater degrees of deformation as compared to White Blood Cells (WBCs). The underlying reason for this distinction lies in the inherent biophysical properties of these cell types. HCT-116 cells are characterized by a lower stiffness^45,46^, which confers upon them a higher degree of flexibility. This malleability allows these cells to adapt their shape more readily in response to external pressures, consequently enabling a swifter transit through narrow spaces. This quality not only enhances their overall velocity but also results in a decreased transition time, albeit at the cost of experiencing greater deformation. The same reasoning can explain the lower maximum velocity of the WBCs due to its reduced deformability. The relative stiffness of WBCs impairs their ability to modify their shape optimally to navigate the physical constraints of the narrow channel. This limitation subsequently slows down their transit time, as they lack the shape flexibility needed to maintain higher velocities.

Advancing from these observations, Figure 2(d) provides a graphical depiction of the correlation matrix in the form of a heatmap, complemented by accompanying p-values. Each p-value is a statistical metric that measures the strength of evidence for rejecting the null hypothesis in the context of evaluated features. Notably, all p-values displayed are less than 0.05, indicating strong evidence against the null hypothesis. This suggests the correlations observed are statistically significant and unlikely due to random chance. Even more compelling are two specific correlations (R1 and R3), with p-values *~* 0, implying an exceedingly strong level of statistical significance. To further investigate the training features, we plotted regression coefficients along with measured error bars. These coefficients provide a quantitative measure of the rate of change in one variable (dependent) due to a one-unit change in another variable (independent). Remarkably, the third relationship (R3), which is between maximum velocity and transient time, showcases a high regression coefficient of *~* 0.96. This high value suggests a robust association between these two variables, a finding that aligns intuitively with our understanding. As expected, the time needed for a cell to traverse through a narrow channel is inversely proportional to its velocity, an increase in velocity leads to a decrease in transit time, hence the strong relationship.

Figure 2(f) presents our implementation of cross-validation, a critical technique in statistical modeling, where we designated four stacks for training and one stack for validation purposes. Cross-validation is fundamental to the robustness of machine learning models as it mitigates overfitting, enabling us to assess how well our model generalizes to unseen data. By partitioning our data into separate training and validation stacks, we can train the model on one set of data and then validate it on a completely separate set. This not only provides an unbiased evaluation of the model’s performance but also ensures that our findings are not mere artifacts of our training data, thus promoting the generalizability and reliability of our model. Furthermore, following the exhaustive cross-validation procedure, we scrutinized the model’s performance utilizing a separate testing dataset. This dataset was not part of either the training or validation phase of the cross-validation process. This evaluation strategy allowed us to further test the model’s performance on unfamiliar data, thereby helping us verify that the model does not produce biased results based on its training and validation data^40,47^.

Our study delved into the exploration of the predictive efficacy of several classification models, specifically Logistic Regression (LR), Support Vector Machine (SVM), Decision Tree (DT), Random Forest (RF), and K-Nearest Neighbors (KNN). Each of these models provides insights, as they are grounded in distinct computational approaches and theoretical underpinnings. LR, for instance, relies on statistical analysis to estimate probabilities, while the SVM employs geometric principles to maximize the margin between classes. DT and RF are built upon hierarchical structures that aim to split data into distinct subsets based on feature characteristics. Lastly, the KNN model classifies new instances based on their proximity to existing instances in the feature space^48,49^.

**Fig. 3:**
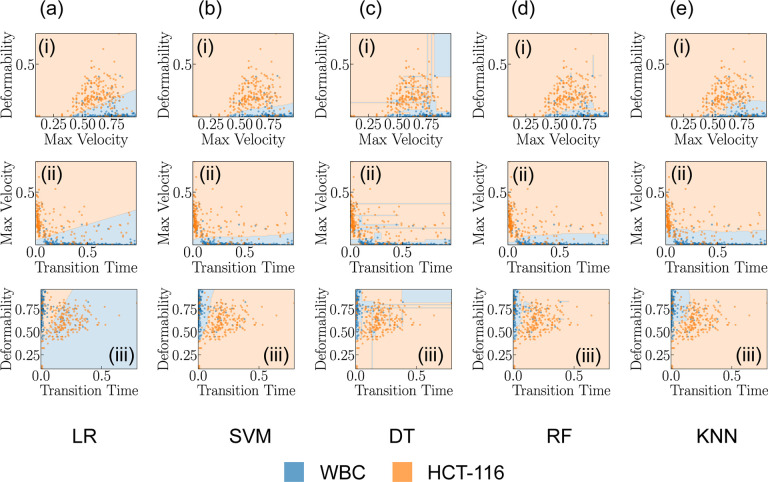
Analysis of Classification Models. (a-e)(i-v) showcases the predictive capabilities of each classification model for varying feature combinations.

**Fig. 4:**
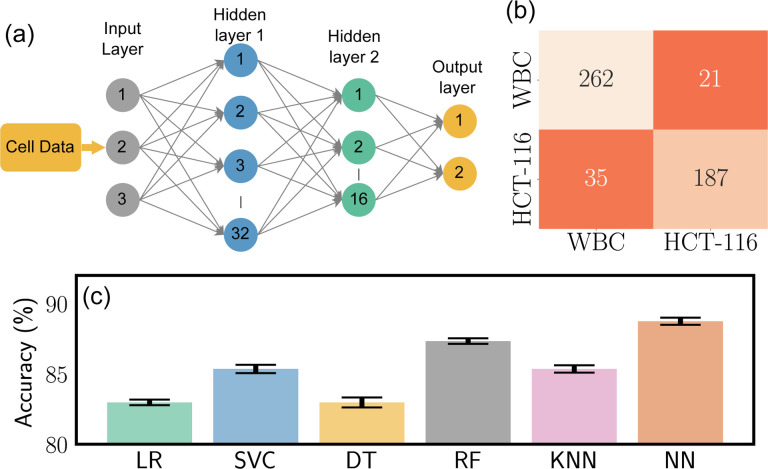
Analysis of Neural Network Model, (a)the architectural design of the neural network dedicated to predicting cell classes, (b) the confusion matrix, outlining the model performance against the testing dataset from the neural network, (c) computed accuracy across all models.

To get a nuanced understanding of the predictive power of these models, we employed each pair of features in our dataset to train these models. The results of these model predictions, excluding those from the Neural Network (NN), are visually represented in Figure 3(a–e)(i–v). This comparative analysis not only helps us understand how different models interpret and predict the relationships between specific feature pairs but also serves as an invaluable resource for refining hyperparameters for model optimization. Through the systematic scrutiny of diverse models and feature combinations, we have obtained a comprehensive perspective of the predictive landscape inherent in our data. We further extended our exploration to the prediction capabilities of NNs. Figure 4(a) delineates the architecture of the NN model that was employed in predicting cell types based on the features previously discussed. This architecture obtained through our ablation study (see supplementary material note 3 for detail) constitutes a simple yet effective network comprising two hidden layers containing 32 and 16 neurons, respectively. The first three input neurons are engaged in processing the input, while the final two neurons are tasked with generating the predicted class. To further quantify the performance of our model, we have also included a confusion matrix in Figure 4(b), which was derived from the testing dataset. A confusion matrix is a critical tool in machine learning that allows for a detailed assessment of a classifier’s performance. It provides insights into not only the model’s accuracy but also the nature of errors it makes, delineating false positives and negatives as well as true positives and negatives. This granular evaluation assists in identifying areas for model improvement and enhances our understanding of its predictive capabilities.

In line with this performance evaluation, Figure 4(c) showcases a comparative analysis of the accuracy of each evaluated model. Accuracy, in this context, refers to the proportion of correct predictions made by the model relative to the total number of test datasets. This measure serves as a fundamental metric in evaluating the overall performance of classification models. From the visual representation, it is evident that the Neural Network (NN) model exhibits best performance compared to the other models we tested. Its demonstrated ability to more accurately classify cell types based on the feature sets used instills confidence in its predictive power. Given its performance, we decided to adopt the NN model for further exploration and classification of cells in our study. This decision is grounded not only in the NN model’s accuracy but also in its capacity for non-linear classification and its inherent ability to learn complex patterns, making it an ideal choice for our subsequent investigation into cell classification.

### 2.3 Detection of cell classes by Convolutional neural network

**Fig. 5:**
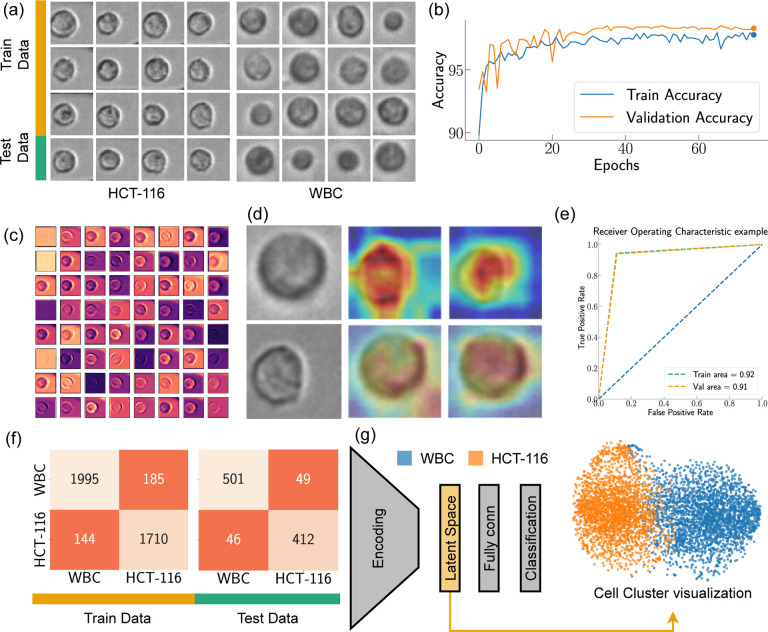
Analysis of Convolution Neural Network(CNN). (a)Sample images from training and validation dataset (b) Training and testing accuracy while training (c) Feature map visualization for WBC (d) Grad-CAM visualization for WBC and HCT-116 (e) the confusion matrix, outlining the model performance against the training and validation dataset from the CNN. (f) illustration of our CNN architecture along with the TSNE plot generated from latent space.

The CNN we used can be divided into two parts: a convolutional feature extraction part (called ‘Encoder’) followed by the fully connected layers classifying the input based on the features (Figure 5(g)). The encoder is structured with an initial convolutional layer, succeeded by four progressive stages, each consisting of multiple blocks. These blocks are critical to the architecture, as they incorporate shortcut connections that perform identity mapping, with their outputs added to the outputs of the stacked layers. The shortcut connection is critical to solving the vanishing gradient problem, a common obstacle encountered during the training of deep NNs^50^. The initial layer is a 7×7 convolutional layer with a stride of 2, followed by batch normalization and a ReLU activation function, and finally max pooling. The four subsequent stages contain two blocks each, with the number of convolutional filters doubling at every stage, beginning from 64 filters in the first stage. Downsampling is performed by convolving with a stride of 2 in the first layer of the 2nd, 3rd, and 4th stages, excluding the shortcut connections, where 1×1 convolutions are applied to match the dimensions. Each of these convolutional layers is succeeded by batch normalization and a ReLU activation function. Following the final stage, there is a global average pooling layer and a fully connected layer that leads to the final classification output. The design of our CNN model, with its specialized blocks and skip connections, provides an efficient way to train deep networks by facilitating the propagation of gradients throughout the entire network. The accuracies obtained from the training and validation datasets have been visualized in Figure 5(b). As the figure illustrates, following 40 epochs of training, we observe a steady fluctuation in accuracy levels. This consistency in fluctuation suggests that the model reaches a relatively stable state of learning after the 40th epoch, indicating diminishing returns from further training. It’s critical to highlight validation accuracy is almost the same as the training accuracy, implying that the model maintained a balance between learning from the training set and generalizing to the validation set. This observation is crucial in asserting the model’s capacity to avoid overfitting, hence providing a reliable and robust solution for the task at hand.

Figure 5(c) offers a detailed graphical representation of the first convolutional layer’s feature map for WBC, incorporating all 64 filters of this layer. This feature map is an integral aspect of our study as it captures the distinctive features that the convolutional layer has learned to identify. Each of the 64 filters in this layer has learned to recognize different characteristics of WBC. For instance, some might be specializing in detecting the contours, some may focus on textural information, while others might be zeroing in on more complex patterns. Visualizing these feature maps allows us to gain an understanding of the underlying mechanics of our model—what exactly it is picking up from the WBC images. By investigating these visualizations, we are essentially interpreting the model’s learning process, which allows refining our model and augmenting its overall performance in the process^51^. Equally important is the role of Gradient-weighted Class Activation Mapping (Grad-CAM) in model interpretability^52^. In Figure 5(d), we illustrate its application, showcasing Grad-CAM for the penultimate and last convolutional layers, with a particular focus on HCT-116 and WBC. The heat maps generated through the Grad-CAM are superimposed over the original images to provide a lucid understanding of the model’s focus during its learning process. From the representation, it is evident that the penultimate convolutional layer is casting a broad net, capturing a substantial amount of background information, yet the primary emphasis remains on the cell structure. This layer acts as a broad filter, capturing both the cell and its surrounding context, which can be critical in many image recognition tasks. As the model progresses to the last layer, it significantly refines its focus. It zeroes in predominantly on the areas of the images that encapsulate the cell, displaying an acute understanding of the cell’s morphology. This targeted approach underscores the layer’s role in the identification of cell types based on their distinct morphological features. For the assessment of our model, we utilized the Receiver Operating Characteristic (ROC) curve, a crucial graphical tool for the evaluation of binary classification models, as illustrated in Figure 5(e). By graphically contrasting the true positive rate (TPR) against the false positive rate (FPR) at various decision thresholds, the ROC curve serves as a potent tool to measure the efficacy of our classification model^53,54^. In this instance, the ROC curves have been generated separately for both the training and testing datasets. Notably, the Area Under the Curve (AUC), a crucial performance metric for the classifier, manifests a remarkable consistency for both our training and validation datasets (Training area *~* testing area = 0.01). This consistency in the AUC values implies that our model exhibits no signs of overfitting - a common complication in machine learning where models tailor themselves too closely to the training data, compromising their capacity to generalize on unseen data. Instead, our model illustrates a balance between learning and generalizability, an unavoidable attribute in practical applications.

Figure 5(f) offers a visualization of the predictions from our trained model in the form of a confusion matrix drawn separately for both the training and validation datasets. The results reveal a commendable level of accuracy for both datasets. The model’s performance on the training data showcases an accuracy of *~* 91.81%, demonstrating its effective learning from the given samples. Concurrently, the model has exhibited commendable performance on the validation dataset, attaining an accuracy of *~* 90.05%. The validation accuracy is of particular importance as it indicates how well our model is likely to perform on unseen, real-world data. The close proximity of these accuracy values suggests a well-balanced model that has avoided overfitting, demonstrating robust learning from the training data while still maintaining the ability to generalize effectively to new data. We extracted the features predicted by our model from the latent space and visualized them via a t-Distributed Stochastic Neighbor Embedding (t-SNE) plot to unfold the high-dimensional data narrative. t-SNE, a robust machine learning algorithm, excels in the visualization of high-dimensional data^55^. It converts the similarities among data points into joint probabilities, endeavoring to minimize the Kullback-Leibler divergence between these joint probabilities in the low-dimensional embedding and the original high-dimensional data. This powerful technique provides a pathway to visualize the high-dimensional data captured in our CNN’s latent space in a 2D format, offering an easily interpretable perspective. In our study, the latent space of our CNN model stored high-dimensional representations of the inputs, which encapsulated the abstract features that the model had learned. Transposing these representations into a t-SNE plot allowed us to take this complex, high-dimensional information and present it in a comprehensible, visually coherent format. Upon inspecting the t-SNE visualization, we observed a notable overlap between the clusters representing White Blood Cells (WBC) and HCT-116. This overlap suggests a visual similarity between these two cell types that the image-based CNN model has difficulty differentiating. This insight emphasizes the limitations of image-only models like CNNs in distinguishing intricate cellular characteristics and underscores the potential need for integrating other forms of data to improve cell differentiation performance.

### 2.4 Multiplex Image machine learning for cell detection

To enhance the accuracy of cell classification, we have developed a novel architectural model, aptly termed the Multiplex Image Machine Learning (MIML) Architecture. This cutting-edge model is capable of processing both image and text data concurrently, enabling it to predict cell types based on a more comprehensive set of input data. The MIML Architecture’s underlying mechanics involve the fusion of image and text features containing cell mechanical properties as described in the results section above. Specifically, after processing the cell images through CNN, the resultant latent space is combined with the output from the traditional NN, which processes the textual information. This integration happens at a Fully Connected (FC) layer, ensuring that the combined features from both modalities are effectively utilized for the final prediction in cell classification. Our MIML model seamlessly integrates a CNN with a traditional NN for advanced performance. The strength of this integrated model lies in its ability to seamlessly handle and interpret both cell images and associated textual information (mechanical properties) regarding cell properties. As such, it encapsulates a broader perspective of cellular data, facilitating a more nuanced understanding and classification of the cells. Our empirical results underline the efficacy of the MIML model, yielding significantly higher accuracy levels in comparison to standalone implementations of the CNN or NN models. This improvement underscores the potential of leveraging multi-modal data – incorporating both image and text – to substantially enhance the performance of cell classification tasks in machine learning applications.

**Fig. 6:**
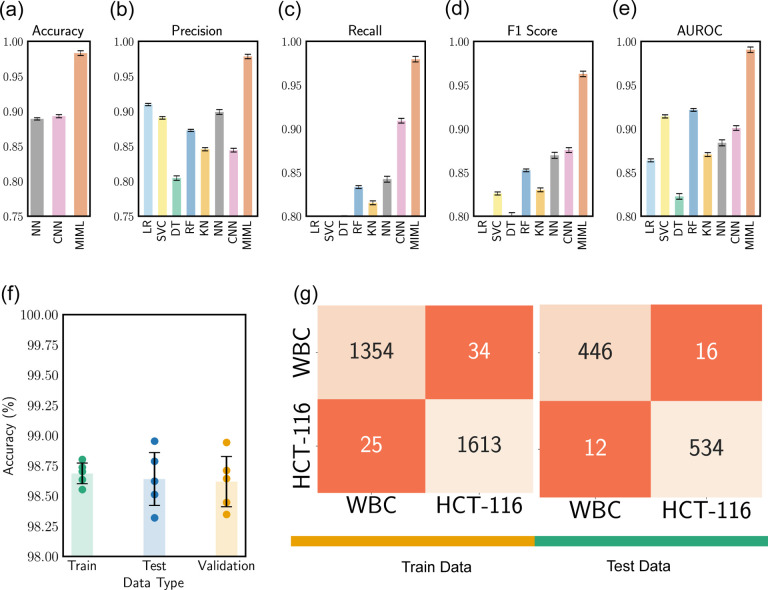
Model Performance Evaluation, (a)-(e) present the comparative assessment of Accuracy, Precision, Recall, F1 score, and Area Under the Receiver Operating Characteristic (AUROC) across various models, (f) showcases the training, testing, and validation accuracy specific to the Multi-Instance Multi-Label (MIML) model, (g) displays the confusion matrix pertaining to both training and testing datasets within the MIML model.

In our exploration of the efficacy of various machine learning models, including the MIML model, we investigate several key performance indicators. Specifically, we focused on metrics such as accuracy, precision, recall, the F1 score, and the area under the Receiver Operating Characteristics (AUROC) as shown in Figure6(a–e). Our evaluation framework was constructed around the aggregate count of true positives (TP), true negatives (TN), false positives (FP), and false negatives (FN) that were recorded during the model’s predictions. These components form the foundation for our performance metrics, and their careful consideration is vital in the detailed dissection of our model’s performance eq.(2–5).

(2) $$\text{Accuracy }=\frac{TP+TN}{TP+TN+FP+FN}$$

(3) $$\text{Precision }=\frac{TP}{TP+FP}$$

(4) $$\text{Recall }=\frac{TP}{TP+FN}$$

(5) $$F1=\left({\text{ Precision }}^{-1}+{\text{ Recall }}^{-1}\right)=\frac{2TP}{2TP+FP+FN}$$

Accuracy is a fundamental measure providing a clear overview by accounting for the overall percentage of correct classifications among all predictions made. While its simplicity is appealing, accuracy might sometimes be deceptive, especially in situations with skewed class distributions. Our model showcases an accuracy improvement of *~*10% compared to pure image-based CNN model. As we delve deeper, we encounter precision, which allows us to zoom into the model’s positive predictions. Also known as the positive predictive value, precision quantifies the fraction of true positive predictions amidst all positive predictions made, a critical indicator when the implications of false positives are substantial. In this regard, our model surpasses CNN with a precision advantage of *~*16%. Our exploration then pivots towards re call or sensitivity, another perspective-shifting metric that focuses on the actual positive cases, computing the proportion that the model correctly identifies. Its criticality surges when the repercussions of false negatives are high, ensuring that the model captures all relevant instances. Our model presents a recall improvement of *~*6.6% over the alternative CNN model. Bridging precision and recall, we have the F1 score. This reconciling metric provides a balanced measure of a model’s performance by combining both precision and recall into a single entity. With its value oscillating between 0 (worst) and 1 (perfect precision and recall), the F1 score offers a comprehensive picture of the model’s performance. Herein, our model boasts an F1 score elevation of *~*10% in relation to the CNN model. Finally, we engage with the Area Under the Receiver Operating Characteristics (AUROC), a metric that transcends individual outcomes to evaluate overall model performance. It represents the probability that the model will rank a randomly chosen positive instance higher than a negative one. The true value of AUROC shines as it evaluates both true positive and false positive rates, offering an all-encompassing performance view across all classification thresholds. Within this sphere, our model achieves an AUROC enhancement of *~*10% compared to its counterpart. By analyzing these interconnected metrics, we elucidate the comprehensive performance profile of our models. Examining Figure 6(a–e), the superior performance of our MIML model is evident across all evaluation categories. This clear edge substantiates the importance of incorporating both image and cellular mechanical properties as inputs, demonstrating the efficacy of our approach. Notably, the significant leap in performance introduced by our MIML model suggests its effectiveness, underscoring a promising advancement in cell classification methodologies.

To provide a more nuanced evaluation of our MIML model, we have devised a composite visualization combining a bar chart Figure 6(f) and a scatter plot. The bar chart portrays the mean accuracies for the training, testing, and validation datasets derived from five-fold cross-validation, with each bar’s height representing the mean accuracy and the attached error bars denoting the variability in the results. Superimposed on this bar chart, we have a scatter plot that displays the individual accuracies from each of the five cross-validation trials. This layered presentation affords a more comprehensive overview of the model’s performance. The close agreement in the training accuracies across cross-validation trials, with an average increase of *~* 10.4% compared to the pure image-based CNN model, reinforces the model’s reproducibility with the training dataset. Simultaneously, the proximity of the validation and testing accuracies to each other, with an average improvement of *~* 10.5% signifies the model’s robust generalizability, suggesting the absence of overfitting. This combined visualization not only confirms the reliability of our MIML model but also offers valuable insights into its performance dynamics.

**Fig. 7:**
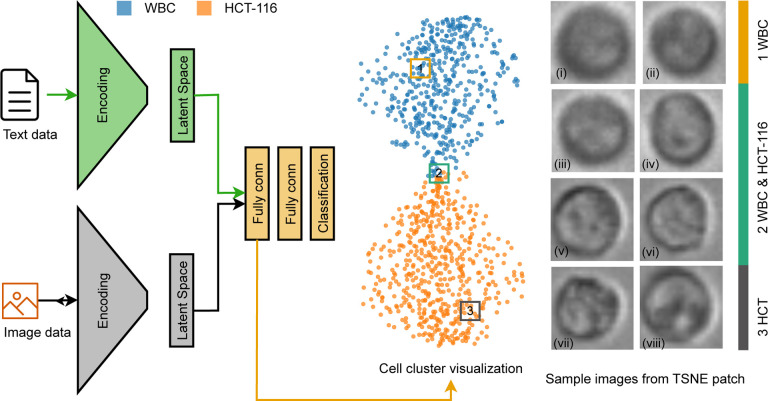
Overview of MIML Cell Classification: (a) Schematic representation of the MIML architecture for cell class prediction, (b) TSNE visualization derived from the latent space, (c)(i-viii) Randomly chosen images from selected patches, with (i-iv) representing WBC and (v-viii) depicting HCT116.

We investigate the diversity of the patterns presented in our data set. To do so, we randomly selected three patches within the latent space and visualized the images associated with them (Figure7(i–viii)). Interestingly, despite an initial impression of high similarity among the images, the model was able to differentiate between them. On a superficial level, all three groups appeared quite alike, with subtle variations only perceptible upon meticulous examination. However, when processed through our model, these seemingly subtle differences were amplified, and the model distinctly categorized each group. Group 2, while almost indistinguishable from the other groups by eye, was identified by the model as possessing specific attributes that set it apart. Similarly, Group 1, and Group 3, despite their visible similarities to each other, were distinctly classified based on the model’s analysis of the underlying patterns and structures in the data. This reveals the power and sensitivity of our model in distinguishing between seemingly identical data points. Even when human observers might struggle to discern any differences due to their apparent similarities, the model is capable of picking up on minute differences and categorizing the data accurately. This affirms the strength of our model in dealing with complex, high-dimensional data and underscores its potential utility in various fields where subtle variations in data could hold significant implications.

## 3 Methods

### 3.1 Preparation of the microfluidic device

The microfluidic channels are produced via the conventional UV lithography technique. Initially, channel designs are drafted using AutoCAD. Using the direct laser writing tool, DWL 66+ (sourced from the Quattrone Nanofabrication Facility at the Singh Center for Nanotechnology, University of Pennsylvania), chrome masks are created. These masks then facilitate the creation of the master pattern on an SU-8 2007 (MicroChem) layer on a silicon wafer, executed at the Center for Photonics and Nanoelectronics (CPN) at Lehigh University. The SU8–2007 is applied to the silicon wafer at a speed of 1000 rpm. Following a soft bake phase, the SU-8 undergoes UV exposure using the Suss MA6/BA6. Post-development, the SU-8 designs undergo a hard bake at 150°C for 30 minutes. Sylgard 184 PDMS, combined with its curing agent at a 10:1 ratio, is poured onto the photoresist master. After a 2-hour degassing period for the PDMS, it is allowed to cure overnight in an oven. Finally, the inlets and outlets are created in the PDMS channel prior to its attachment to a large coverslip, secured with oxygen plasma treatment.

### 3.2 Cell culture and data collection

The Human Colorectal Cancer cell line (HCT116), which was purchased from American Type Culture Collection (ATCC), and cultured in Dulbecco’s Modified Eagle’s Medium (DMEM, Gibco). The DMEM was meticulously supplemented with 10% Fetal Bovine Serum (FBS, Gibco), and 100 U/mL of Penicillin Streptomycin (R&D system. To maintain the cells in an optimal state, we change the culture medium every other day. To passage or isolate to single cell, we employed a 0.05% Trypsin-EDTA (Bio-Techne Corporation. The Human Peripheral Blood Mononuclear cells (PBMCs) were obtained from the Human Immunology Core at Penn Medicine. These PBMCs were maintained in RPMI-1640 medium (ATCC), fortified with 10% FBS and 100 U/mL of Penicillin-Streptomycin. All the bright field images were captured using Nikon Eclipse TE2000S inverted microscope with a Ximea CCD Camera. All the images were taken with the same setting for comparison.

### 3.3 Feature extraction

During our investigation, we implemented an experimental protocol that guided the cells through carefully constructed, narrow passages. Our ability to detect and scrutinize these cells was enhanced by a straightforward yet potent machine learning model - Yolov5. This model generated bounding boxes, a crucial tool that helped us concentrate on each individual cell, even when they were under the pressure of induced deformation. Within these defined areas, we initiated a detailed analysis of several parameters. As a first step, we computed the deformation index, a metric indicating the degree of cellular deformation. Concurrently, we methodically measured the time taken by each cell to travel the entire length of the channel, a metric defined as Transition Time 1(c). We also evaluated the maximum velocity achieved by each cell during its passage through the channel. Upon calculating the aforementioned parameters, we segmented the cell image and stored it along with its measured property for subsequent training of our MIML model.

## 4 Conclusion

In this study, we introduce a novel machine learning architecture, which we refer to as MIML, specifically designed to integrate label-free cell imagery with cell biomechanical property data. This innovative combination harnesses the full potential of the morphological information intrinsic to each cell, which is often overlooked or discarded in conventional machine learning approaches. Our primary objective with MIML is to enhance the precision of cell classification by broadening the range of cell characteristics considered. The MLML architecture synergistically combines these two data types to create a comprehensive cell profile. By integrating both label-free images and biomechanical data, our model provides a more holistic understanding of the cell. It can capture and utilize the rich morphological information usually discarded by traditional machine learning models. As a result, we have achieved a higher accuracy rate in cell classification compared to models that only consider one type of data. Furthermore, this methodology bears extensive implications across disciplines such as disease diagnostics and the deciphering of cell behavior, laying the groundwork for the creation of enhanced therapeutic tactics and treatment regimes. This model’s application has been demonstrated in a use-case scenario involving the classification of White Blood Cells (WBCs) and HCT116 cells. Nevertheless, the inherent versatility of the model, facilitated by transfer learning, allows for its potential deployment in the categorization of any cellular species. This is particularly effective when the cells are morphologically similar yet possess distinct biomechanical properties, thus extending the model’s applicability beyond its initial design.

## Supplementary Material

1
