# [WITHDRAWN] MIML: Multiplex Image Machine Learning for High Precision Cell Classification via Mechanical Traits within Microfluidic Systems

**Published:** 2024-01-24

**Authors:** Khayrul Islam, Ratul Paul, Shen Wang, Yaling Liu

## Abstract

This paper has been withdrawn by Khayrul Islam.

