# Supplementary material for: MIML: Multiplex Image Machine Learning for High Precision Cell Classification via Mechanical Traits within Microfluidic Systems
Source: ArXiv. 2025 Feb 24:arXiv:2309.08421v3. Originally published 2023 Sep 15. Preprint. [Version 3] (PMC10516114)
Supplement: Supplement 1 [file NIHPP2309.08421v3-supplement-1.pdf]

Supplementary Material:

Supplementary note 1

Introduction to cell sorting methods

Traditional cell sorting methodologies can be classified into two categories: bulk sorters, which include Magnetic-Activated Cell Sorting (MACS)[1,2], filtration-based methods[3–5], Deterministic Lateral Displacement[6,7], and optical lattices[8], and single-cell sorters like Fluorescence-Activated Cell Sorters (FACS)[9–11] and light scattering- and image-based flow cytometers[12–14].

Bulk sorters offer the advantage of high-throughput operation due to their massively parallel approach, separating cells passively based on a small number of predetermined parameters. However, this category of sorters lacks the flexibility to separate individual cells based on variable choice of orthogonal features, such as fluorescence, size, and deformation, within a single sorting run.

On the other hand, single-cell sorters, albeit slower due to their sequential operation, provide a higher degree of flexibility. They allow for cell-specific sorting decisions based on a large array of flexibly chosen features. Both these categories largely depend on cell identification, and, in the case of MACS, cell separation based on molecular labels. Although this provides specificity, it has potential drawbacks such as altering cellular function, increased cost and preparation time, and could limit the identification of cell subpopulations without known molecular markers. Furthermore, this reliance may prove incompatible with certain subsequent uses such as transplantation.

Label-free identification of cells offers an alternative approach with its benefits (see supplementary note 2 drawback of fluorescent labeling), an exemplary method being the usage of forward and side scatter signals in flow cytometry. This method, although indirect, provides information on the size and internal structure of cells. Several label-free approaches focus on different chemical or physical properties of cells. For instance, Raman scattering provides multiplexed data on the presence of chemical species in cells, quantitative phase imaging offers internal mass density distributions, and techniques like Brillouin scattering and deformability cytometry are employed for mechanical phenotyping of cells.

Label-free sorting of cells, using parameter-based or AI-based approaches, is highly desirable for various downstream applications. Our method employs an innovative strategy that combines both visual data (cell images) and biomechanical properties to classify cells that are visually indistinguishable. This multi-modal analysis provides a more comprehensive profile of the cells under study and aids in distinguishing between cell types that share similar visual characteristics but exhibit differences at the mechanical level.

Supplementary note 2

Ablation study for NN model

We conducted a comprehensive ablation analysis to systematically evaluate the performance of our neural network model under varying configurations. Our investigation primarily focuses on alterations to the hidden layers and neurons within the network, offering an understanding of the impact these parameters have on our NN model accuracy. We examined three distinct layers within the neural network, manipulating the quantity of neurons in each. Specifically, we explored configurations encompassing 1 to 3 layers, each hosting a varying number of neurons 16, 32, and 64, respectively. This manipulation resulted in a total of ten unique configurations that were subsequently assessed (Table 1).

Model name	Layer 1	Layer 2	Layer 3	Accuracy
**M1**	16	x	x	81.97
**M2**	32	x	x	82.35
**M3**	64	x	x	82.97
**M4**	16	16	x	84.33
**M5**	32	32	x	86.35
**M6**	64	64	x	86.74
**M7**	32	16	x	88.74
**M8**	64	32	x	89.05
**M9**	32	64	32	88.67
**M10**	16	32	16	87.97

Table 1: Accuracy Results for Various Configurations in the Neural Network Model Ablation Study

Each configuration was subjected to the same training and testing procedures, maintaining consistency across the study. The performance was evaluated using a unified metric of accuracy, enabling us to directly compare the outcomes of each configuration. This ablation study yielded significant insights into the optimal structure of the neural network for our specific task. The results demonstrated the influence of the number of hidden layers and the number of neurons per layer on the overall performance of the neural network.

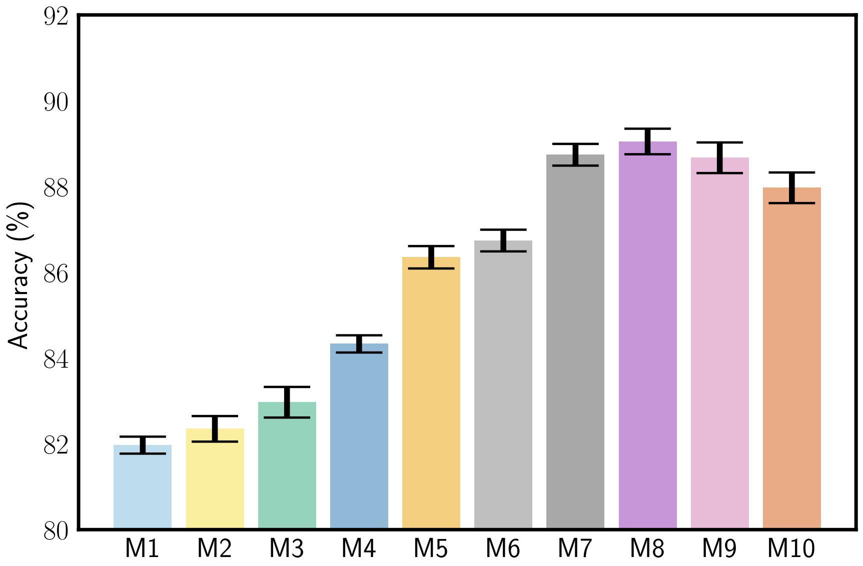

Figure S1: Bar Chart of Model Accuracies from the Ablation Study

The results of our investigation suggest an encouraging trend wherein model accuracy demonstrates a consistent increase in line with the complexity of the model up to M9. Nevertheless, our selection favored model M7 over M8. Although M8 exhibited marginally higher performance, the difference in accuracy was not significant. Crucially, model M7 achieves comparable performance whilst necessitating approximately 30% fewer trainable parameters. This makes M7 a more resource-efficient choice, striking an optimal balance between model complexity, performance, and computational efficiency.

Supplementary note 3

Cell Diameter and Deformation Index Analysis

To assess the potential influence of cell diameter on the deformation index (DI), we analyzed the relationship between cell size and DI for the two cell types used in this study: HCT116 and WBC. This analysis aimed to evaluate whether cell size significantly contributes to the differences observed in DI, a key feature for cell classification in our framework.

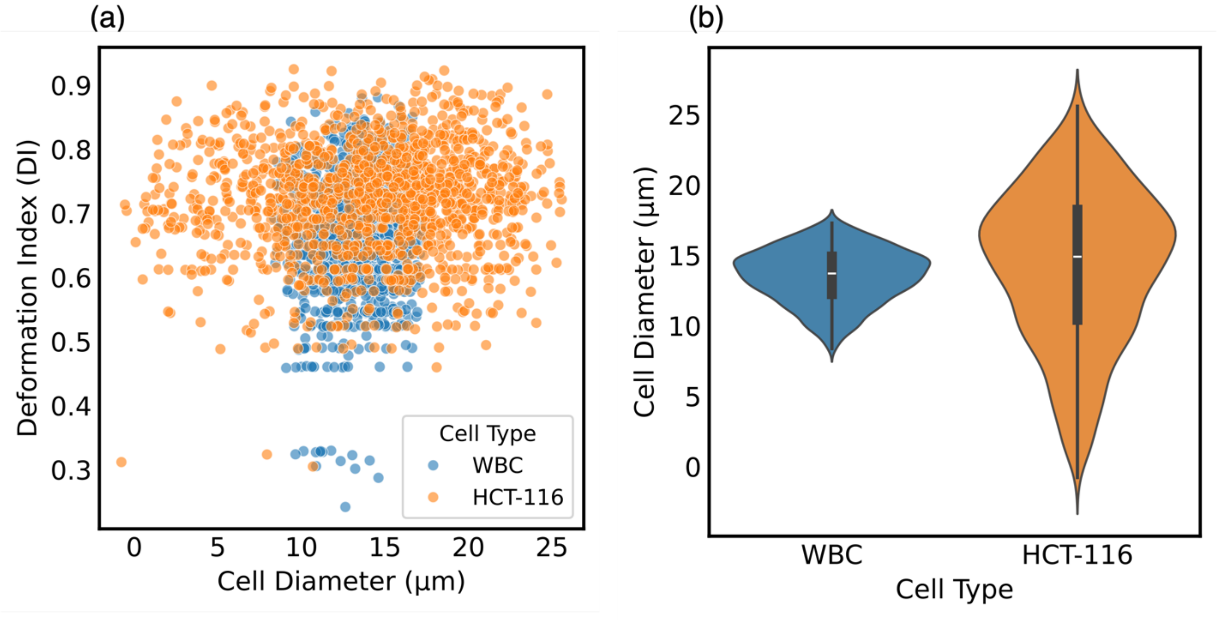

Figure S2: Cell Diameter and Deformation Index Analysis, (a) Cell diameter vs DI, (b) Violine plot of cell diameter

Figure S2(a) shows the scatterplot of DI versus cell diameter for each cell type. Correlation analysis indicates weak positive correlations for both HCT116 cells (correlation = 0.06, p-value = 0.025) and WBC cells (correlation = 0.09, p-value = 0.002). These low correlation values suggest that cell size has only a minor influence on DI. Instead, DI is predominantly determined by the intrinsic mechanical properties of the cells. Figure S2(b) depicts violin plots of the cell diameter distributions for HCT116 and WBC cells. The mean diameter of HCT116 cells is 14.2 µm (± 5.48 µm), while WBC cells have a mean diameter of 13.5 µm (± 1.91 µm). Although HCT116 cells exhibit a broader range of diameters, the overlap between the two cell types indicates that cell size alone does not account for the observed DI differences.

References

1. Miltenyi, S., M"uller, W., Weichel, W. & Radbruch, A. High gradient magnetic cell separation with MACS. *Cytometry* **11**, 231–238 (1990).
2. Šafařík, I. & Šafaříková, M. Use of magnetic techniques for the isolation of cells. *J. Chromatogr. B Biomed. Sci. Appl.* **722**, 33–53 (1999).

3. Preira, P. & Others. Passive circulating cell sorting by deformability using a microfluidic gradual filter. *Lab Chip* **13**, 161–170 (2013).
4. Wang, G. & Others. Microfluidic cellular enrichment and separation through differences in viscoelastic deformation. *Lab Chip* **15**, 532–540 (2015).
5. Cheng, Y., Ye, X., Ma, Z., Xie, S. & Wang, W. High-throughput and clogging-free microfluidic filtration platform for on-chip cell separation from undiluted whole blood. *Biomicrofluidics* **10**, (2016).
6. Huang, L. R., Cox, E. C., Austin, R. H. & Sturm, J. C. Continuous Particle Separation Through Deterministic Lateral Displacement. *Science* **304**, 987–990 (2004).
7. Beech, J. P., Holm, S. H., Adolfsson, K. & Tegenfeldt, J. O. Sorting cells by size, shape and deformability. *Lab Chip* **12**, 1048–1051 (2012).
8. MacDonald, M. P., Spalding, G. C. & Dholakia, K. Microfluidic sorting in an optical lattice. *Nature* **426**, 421–424 (2003).
9. Bonner, W. A., Hulett, H. R., Sweet, R. G. & Herzenberg, L. A. Fluorescence activated cell sorting. *Rev. Sci. Instrum.* **43**, 404–409 (1972).
10. Schmid, L., Weitz, D. A. & Franke, T. Sorting drops and cells with acoustics: Acoustic microfluidic fluorescence-activated cell sorter. *Lab Chip* **14**, 3710–3718 (2014).
11. Nawaz, A. A. & Others. Acoustofluidic fluorescence activated cell sorter. *Anal* **87**, 12051–12058 (2015).
12. Shapiro, H. M. *Practical Flow Cytometry*. (Wiley & Sons, Incorporated, John, 2005).
13. Nitta, N. & Others. Intelligent Image-Activated Cell Sorting. *Cell* **175**, 266–276 (2018).
14. Ota, S. & Others. Ghost cytometry. *Science* **360**, 1246–1251 (2018).
15. Progatzky, F., Dallman, M. J. & Lo Celso, C. From seeing to believing: labelling strategies for in vivo cell-tracking experiments. *Interface Focus* **3**, 001 (2013).
16. Li, P. & Others. Sheathless acoustic fluorescence activated cell sorting (aFACS) with high cell viability. *Anal* **91**, 15425–15435 (2019).

17. Mikulová, V., Kološtová, K. & Zima, T. Methods for detection of circulating tumour cells and their clinical value in cancer patients. *Folia Biol.* **57**, 151–161 (2011).

18. Ettinger, A. & Wittmann, T. Fluorescence live cell imaging. *Methods Cell Biol.* **123**, 77–94 (2014).

19. Cossarizza, A. & Others. Guidelines for the use of flow cytometry and cell sorting in immunological studies (third edition). *Eur* **51**, 2708–3145 (2021).

20. Basiji, D. A., Ortyn, W. E., Liang, L., Venkatachalam, V. & Morrissey, P. Cellular image analysis and imaging by flow cytometry. in *Clin* 653–670 (Lab. Med. 27, 2007).

21. Chen, D., Lackner, M. & Punnoose, E. *Molecular biomarker analyses using circulating tumor cells Atwal SK*.
