# Supplementary material for: MIML: Multiplex Image Machine Learning for High Precision Cell Classification via Mechanical Traits within Microfluidic Systems
Source: ArXiv. 2023 Sep 15:arXiv:2309.08421v1. Preprint. [Version 1] (PMC10516114)
Supplement: Supplementary file 1 [file NIHPP2309.08421V1-supplement-1.pdf]

Supplementary Material:

Supplementary note 1

Introduction to cell sorting methods

Traditional cell sorting methodologies can be classified into two categories: bulk sorters, which include Magnetic-Activated Cell Sorting (MACS)[1,2], filtration-based methods[3–5], Deterministic Lateral Displacement[6,7], and optical lattices[8], and single-cell sorters like Fluorescence-Activated Cell Sorters (FACS)[9–11] and light scattering- and image-based flow cytometers[12–14].

Bulk sorters offer the advantage of high-throughput operation due to their massively parallel approach, separating cells passively based on a small number of predetermined parameters. However, this category of sorters lacks the flexibility to separate individual cells based on variable choice of orthogonal features, such as fluorescence, size, and deformation, within a single sorting run.

On the other hand, single-cell sorters, albeit slower due to their sequential operation, provide a higher degree of flexibility. They allow for cell-specific sorting decisions based on a large array of flexibly chosen features. Both these categories largely depend on cell identification, and, in the case of MACS, cell separation based on molecular labels. Although this provides specificity, it has potential drawbacks such as altering cellular function, increased cost and preparation time, and could limit the identification of cell subpopulations without known molecular markers. Furthermore, this reliance may prove incompatible with certain subsequent uses such as transplantation.

Label-free identification of cells offers an alternative approach with its benefits (see supplementary note 2 drawback of fluorescent labeling), an exemplary method being the usage of forward and side scatter signals in flow cytometry. This method, although indirect, provides information on the size and internal structure of cells. Several label-free approaches focus on different chemical or physical properties of cells. For instance, Raman scattering provides multiplexed data on the presence of chemical species in cells, quantitative phase imaging offers internal mass density distributions, and techniques like Brillouin scattering and deformability cytometry are employed for mechanical phenotyping of cells.

Label-free sorting of cells, using parameter-based or AI-based approaches, is highly desirable for various downstream applications. Our method employs an innovative strategy that combines both visual data (cell images) and biomechanical properties to classify cells that are visually indistinguishable. This multi-modal analysis provides a more comprehensive profile of the cells under study and aids in distinguishing between cell types that share similar visual characteristics but exhibit differences at the mechanical level.

Supplementary note 2

Drawbacks of florescent-based sorting

Existing cell selection procedures utilized for biomedical analysis primarily depend on fluorescence-activated cell sorting (FACS) systems. These systems rely heavily on fluorescent biomarkers and/or the intensity of light scattering[11]. However, current FACS systems exhibit a few significant limitations. They are complex and costly, have a large footprint, and introduce potential risks related to sample contamination and biosafety due to aerosol generation in an open environment[15,16].

Additionally, the fluorescence labeling technique, which is foundational to FACS, has its own set of complications. Firstly, the method is largely dependent on antibody-based fluorescence probes, which are contingent upon the overexpression of certain proteins on the cell membranes. This overexpression is unstable and largely dictated by the type of cancer and the condition of the patient[17]. Secondly, the phenomena of photobleaching and phototoxicity occur in a short duration following exposure to a fluorescent light source, and the selection of suitable fluorophores requires expert knowledge. Thirdly, the process of fluorescence staining negatively impacts the viability of cells, making it disadvantageous for further cell culture and post-processing activities[18,19].

Another significant limitation of conventional FACS is its inability to process high-resolution data such as clear microscopy images, which are critical for the smart and logical selection of cells, particularly rare cells and cells without established or unique biomarkers[20]. For instance, epithelial cell adhesion molecule (EpCAM) is a well-recognized marker for epithelial tumor cells, and anti-EpCAM antibodies have been widely employed for circulating tumor cell (CTC) capture. However, about 20% of the 50 primary types of breast cancer cell lines (representative of luminal, HER2 amplified, and basal-like breast cancers) exhibit little to no EpCAM expression[21].

Therefore, the constraints presented by a classification system based solely on biomarker antibodies are substantial, and the addition of identification criteria could further enhance the efficiency of CTC isolation, enabling regular disease analysis. Dependence on molecular labels might modify the cells and their function, lead to additional costs and preparation time, and could render researchers unaware of cells without known molecular markers or yet unidentified subpopulations. This could also result in incompatibility with future uses, such as transplantation.

Supplementary note 3

Ablation study for NN model

We conducted a comprehensive ablation analysis to systematically evaluate the performance of our neural network model under varying configurations. Our investigation primarily focuses on alterations to the hidden layers and neurons within the network, offering an understanding of the

impact these parameters have on our NN model accuracy. We examined three distinct layers within the neural network, manipulating the quantity of neurons in each. Specifically, we explored configurations encompassing 1 to 3 layers, each hosting a varying number of neurons 16, 32, and 64, respectively. This manipulation resulted in a total of ten unique configurations that were subsequently assessed (Table 1). Each configuration was subjected to the same training and testing procedures, maintaining consistency across the study. The performance was evaluated using a unified metric of accuracy, enabling us to directly compare the outcomes of each configuration. This ablation study yielded significant insights into the optimal structure of the neural network for our specific task. The results demonstrated the influence of the number of hidden layers and the number of neurons per layer on the overall performance of the neural network.

Model name	Layer 1	Layer 2	Layer 3	Accuracy
**M1**	16	x	x	81.97
**M2**	32	x	x	82.35
**M3**	64	x	x	82.97
**M4**	16	16	x	84.33
**M5**	32	32	x	86.35
**M6**	64	64	x	86.74
**M7**	32	16	x	88.74
**M8**	64	32	x	89.05
**M9**	32	64	32	88.67
**M10**	16	32	16	87.97

Table 1: Accuracy Results for Various Configurations in the Neural Network Model Ablation Study

The results of our investigation suggest an encouraging trend wherein model accuracy demonstrates a consistent increase in line with the complexity of the model up to M9. Nevertheless, our selection favored model M7 over M8. Although M8 exhibited marginally higher performance, the difference in accuracy was not significant. Crucially, model M7 achieves comparable performance whilst necessitating approximately 30% fewer trainable parameters. This makes M7 a more resource-efficient choice, striking an optimal balance between model complexity, performance, and computational efficiency.

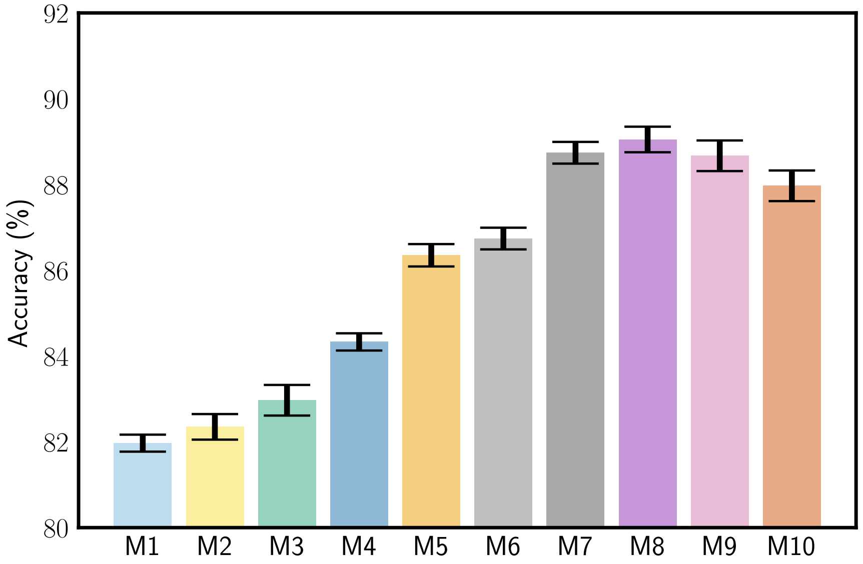

Figure S1: Bar Chart of Model Accuracies from the Ablation Study

References

1. Miltenyi, S., M"uller, W., Weichel, W. & Radbruch, A. High gradient magnetic cell separation with MACS. *Cytometry* **11**, 231–238 (1990).
2. Šafařík, I. & Šafaříková, M. Use of magnetic techniques for the isolation of cells. *J. Chromatogr. B Biomed. Sci. Appl.* **722**, 33–53 (1999).
3. Preira, P. & Others. Passive circulating cell sorting by deformability using a microfluidic gradual filter. *Lab Chip* **13**, 161–170 (2013).
4. Wang, G. & Others. Microfluidic cellular enrichment and separation through differences in viscoelastic deformation. *Lab Chip* **15**, 532–540 (2015).
5. Cheng, Y., Ye, X., Ma, Z., Xie, S. & Wang, W. High-throughput and clogging-free microfluidic filtration platform for on-chip cell separation from undiluted whole blood. *Biomicrofluidics* **10**, (2016).

6. Huang, L. R., Cox, E. C., Austin, R. H. & Sturm, J. C. Continuous Particle Separation Through Deterministic Lateral Displacement. *Science* **304**, 987–990 (2004).
7. Beech, J. P., Holm, S. H., Adolfsson, K. & Tegenfeldt, J. O. Sorting cells by size, shape and deformability. *Lab Chip* **12**, 1048–1051 (2012).
8. MacDonald, M. P., Spalding, G. C. & Dholakia, K. Microfluidic sorting in an optical lattice. *Nature* **426**, 421–424 (2003).
9. Bonner, W. A., Hulett, H. R., Sweet, R. G. & Herzenberg, L. A. Fluorescence activated cell sorting. *Rev. Sci. Instrum.* **43**, 404–409 (1972).
10. Schmid, L., Weitz, D. A. & Franke, T. Sorting drops and cells with acoustics: Acoustic microfluidic fluorescence-activated cell sorter. *Lab Chip* **14**, 3710–3718 (2014).
11. Nawaz, A. A. & Others. Acoustofluidic fluorescence activated cell sorter. *Anal* **87**, 12051–12058 (2015).
12. Shapiro, H. M. *Practical Flow Cytometry*. (Wiley & Sons, Incorporated, John, 2005).
13. Nitta, N. & Others. Intelligent Image-Activated Cell Sorting. *Cell* **175**, 266–276 (2018).
14. Ota, S. & Others. Ghost cytometry. *Science* **360**, 1246–1251 (2018).
15. Progatzky, F., Dallman, M. J. & Lo Celso, C. From seeing to believing: labelling strategies for in vivo cell-tracking experiments. *Interface Focus* **3**, 001 (2013).
16. Li, P. & Others. Sheathless acoustic fluorescence activated cell sorting (aFACS) with high cell viability. *Anal* **91**, 15425–15435 (2019).
17. Mikulová, V., Kološtová, K. & Zima, T. Methods for detection of circulating tumour cells and their clinical value in cancer patients. *Folia Biol.* **57**, 151–161 (2011).
18. Ettinger, A. & Wittmann, T. Fluorescence live cell imaging. *Methods Cell Biol.* **123**, 77–94 (2014).
19. Cossarizza, A. & Others. Guidelines for the use of flow cytometry and cell sorting in immunological studies (third edition). *Eur* **51**, 2708–3145 (2021).

20. Basiji, D. A., Ortyn, W. E., Liang, L., Venkatachalam, V. & Morrissey, P. Cellular image analysis and imaging by flow cytometry. in *Clin* 653–670 (Lab. Med. 27, 2007).

21. Chen, D., Lackner, M. & Punnoose, E. *Molecular biomarker analyses using circulating tumor cells Atwal SK*.
